# Dermatoglyphic patterns as predictors of dental pain perception

**DOI:** 10.6026/973206300223758

**Published:** 2026-06-30

**Authors:** Charanya Chandrasekaran, Harishma S, Renin Maria A, Kokila Sivakumar, Solomon Prabhu V, Nandhini G

**Affiliations:** 1 Department of Conservative Dentistry and Endodontics, Chettinad dental college and research institute, Kelambakkam, Chennai, India; 2 Department of Oral and Maxillofacial Pathology, Adhiparasakthi Dental College and Hospital, Melmaruvathur, Tamil Nadu, India

**Keywords:** Dermatoglyphics, pain perception, biomarkers, pulpitis, visual analogue scale

## Abstract

Pain perception in an individual in endodontics remains a challenge and dermatoglyphics suggests reliable non-invasive markers are lacking. Therefore, it is of interest to evaluate the association between pain and dermatoglyphics from 200 patients within 18 to 45 years, diagnosed with symptomatic irreversible pulpitis and symptomatic apical periodontitis assessed using the Heft-Parker Visual Analogue Scale. The arch pattern demonstrated a significantly higher pain score (n=63) (118.84 ± 30.31) compared with whorl (n=57) (93.29 ± 33.01) and loop (n=80) (93.16 ± 32.36) (F=4.183, p=0.020). The arch pattern was associated with the highest pain, suggesting that dermatoglyphic configurations may serve as non-invasive predictors of individual pain thresholds. Thus, data shows the dermatoglyphics association with endodontic pain perception, thereby proving it to be non-invasive approach for predicting individual pain response before treatment.

## Background:

Endodontic pain, among dental procedures, whether pre-operative or post-operative continues to be one of the most distressing experiences for patients and significantly influences treatment outcomes and patient satisfaction [1]. Pain perception, however, it is inherently subjective and demonstrates considerable inter-individual variability, this variability is influenced by a dynamic interplay of biological, psychological and environmental variables, making the prediction and management of pain particularly challenging in endodontic practice [2]. Despite standardized anesthetic protocols and treatment procedures, patients often exhibit markedly different pain responses, highlighting the need for reliable predictors that can facilitate individualized pain management strategies [2]. One such emerging field is dermatoglyphics, characterized by genetically determined ridge patterns that remain stable throughout life, with minimal influence from post-natal environmental factors [3]. Both dermal ridge patterns and the neural tube originate from the same ectodermal layer during embryogenesis, particularly involving neural crest cells; this shared development origin suggests that dermatoglyphic configurations may reflect underlying neurodevelopment characteristics, including variations in neural processing and sensory perception [4]. Previous studies have demonstrated an association between dermatoglyphic patterns and a range of systemic and oral conditions, including neurodevelopmental disorders, cardiovascular diseases, diabetes, dental caries, malocclusion and periodontal diseases [5]. These findings support the concept that dermatoglyphics may serve as a non-invasive biomarker of genetic predisposition and psychological variability that remains largely unexplored [6]. Currently, pain assessment in dentistry relies predominantly on the subjective tools such as the Visual Analogue Scale (VAS), which, although widely used, are limited to their subjective and retrospective nature [7]. Therefore, it is of interest to describe the association between dermatoglyphic ridge patterns and pain perception amongst patients with endodontic pathology in the Kanchipuram population.

## Materials and Methods:

This cross-sectional observational study was conducted after obtaining approval from the institutional ethical committee (IHEC-CDCRI/2024/STU-0034). Following the approval, the study was conducted according to the principles of the Declaration of Helsinki and written informed consent was obtained from all the participants prior to their enrollment in the study. Based on prior research and statistical power calculations, the study includes a sample size of 200 patients to achieve adequate statistical power of 80% and confidence level of 95%, significance level of 5% to detect a significant correlation between dermatoglyphic patterns and pain scores. A total of two hundred patients aged 18 to 45 years old, diagnosed with symptomatic irreversible pulpitis and symptomatic apical periodontitis following evaluation by thermal testing, were included in the study. Patients with systemic conditions affecting pain perception, those under analgesic or sedative medications, those with dermatological conditions affecting fingerprints and those previously treated for endodontic cases were excluded from the study. The pain scale was evaluated using the Heft - Parker visual analogue scale (HP-VAS) Score. Participants were instructed to indicate their pain level using the scale and the responses were recorded numerically for analysis. The thumbprint pattern was taken after cleaning both hands using an antiseptic solution. The right and left thumbs were firmly pressed on the stamp pad and placed on the A4 sheet. The fingerprints were evaluated using a magnifying lens for various whorl, radial loop and arch patterns (Figure 1 - see PDF). All recordings and analyses were performed by a single calibrated examiner to minimize inter-observer variability. Statistical software such as IBM SPSS Statistics, Version 27 (IBM Corp., Armonk, NY, USA) was used for data analysis. Descriptive statistics were calculated for all the variables. One-way ANOVA was used to analyze the association between pain scores and dermatoglyphic patterns. Post hoc analyses were performed, where appropriate, to identify differences between groups. Multivariate analysis was conducted to control for potential confounding variables, including age, gender and diagnosis. Confidence intervals of 95% and p-value <0.05 was considered to determine the significance of results.

## Results:

A total of 200 participants were included in the study, with dermatoglyphic patterns distributed as loops (n = 80), arches (n = 63) and whorls (n = 57) (Table 1). The mean pain score differed across the three dermatoglyphic groups. Participants with arch patterns demonstrated the highest mean pain score (118.84±30.31), followed by the whorl pattern (93.29±33.01) and loop pattern (93.17±32.37). One-way analysis of variance (ANOVA) revealed a statistically significant difference in the mean pain scores among the three groups (F=4.183, p=0.020). The 95% confidence intervals for the mean pain scores were 104.23-133.45 for the arch pattern, 76.32-110.27 for the whorl pattern and 79.50-106.83 for the loop pattern. The post-hoc analysis demonstrated that the arch pattern groups had significantly higher pain scores compared to both loop and whorl (p<0.05), while no significant difference was observed between loop and whorl patterns.

## Discussion:

Pain perception in endodontics is a complex and multifactorial phenomenon influenced by biological, psychological and neurophysiological factors [1]. Despite standardized clinical protocols, considerable inter-individual variability in pain response is frequently observed, emphasizing the need for reliable predictors of pain sensitivity [3]. Recent studies highlight that pain perception is modulated by both peripheral nociceptive mechanisms and central neural processing, including neuroplastic changes and sensitization pathways [8]. The present study explored the association between dermatoglyphic patterns and endodontic pain perception based on the premise that both epidermal ridge patterns and the central nervous system share a common ectodermal origin during embryogenesis. Dermatoglyphics has been increasingly investigated as a non-invasive biomarker reflecting genetically determined traits, with recent systematic reviews demonstrating its association with various oral and systemic conditions [9, 10]. The embryological linkage between dermal ridge development and neural tissue formation, particularly through neural crest cells, provides a plausible biological basis for its association with neurophysiological responses, including pain perception therefore indirectly reflect differences in neural processing, autonomic regulation and nociceptive sensitivity. Emerging evidence suggests that altered sensory processing and neurophysiological dysregulation play a significant role in pain variability across individuals [11]. The findings of the present study demonstrated a statistically significant association between dermatoglyphic patterns and pain scores, with individuals exhibiting arch patterns reporting the highest pain intensity. This observation suggests that simpler ridge configurations may be associated with heightened nociceptive sensitivity. One possible explanation is that arch patterns, characterized by minimal ridge complexity and absence of triradii, may correlate with reduced autonomic adaptability and altered neural modulations of pain. Contemporary research supports the concept that differences in central sensitization and neural responsiveness significantly influence pain perception [8]. In contrast, loop and whorl patterns, which demonstrate greater structural complexity, may reflect a more balanced neurophysiological response, resulting in comparatively lower pain perception. Studies investigating sensory reactivity and pain modulations have shown that individual variability in neural processing can significantly alter pain thresholds and responses [12]. These findings are consistent with emerging evidence suggesting that dermatoglyphic traits can serve as indicators of underlying genetic and neurodevelopmental influences [13]. Recent cross-sectional and systematic studies have demonstrated an association between dermatoglyphic patterns and oral diseases such as periodontal diseases and caries, reinforcing their role as potential biological markers [8, 13]. However, literature specifically correlating dermatoglyphic patterns with pain perception in endodontics remains limited. The present study, therefore, contributes novel evidence to this evolving field by demonstrating a potential relationship between fingerprint patterns and individual pain response during endodontic conditions. From clinical perspective, the ability to predict pain sensitivity using a simple, non-invasive tool such as dermatoglyphic analysis could have significant implications. Recent studies in dental pain research emphasize the importance of understanding psychoemotional and physiological factors influencing pain perception during dental procedures [14]. Pre-operative identification of patients with a higher predisposition to pain may allow clinicians to adopt tailored pain management strategies, including modification of anaesthetic techniques, pre-emptive analgesia and enhanced patient counselling. Such an approach aligns with the principles of personalized dentistry and precision medicine. Despite these promising findings, the present study has certain limitations. The cross-sectional design limits the ability to establish a causal relationship between dermatoglyphic patterns and pain perception. Pain assessment was based on a subjective scale, which may be influenced by psychological and behavioral factors. Recent literature also highlights that variables such as age, emotional state and cognitive processing can significantly influence pain perception and reporting [15]. Furthermore, the study population was confined to a specific geographic region, which may limit the generalizability of the findings. Longitudinal investigation should focus on larger, multicentric studies incorporating a diverse population to validate these findings. Integration of psychological assessment tools and advanced statistical models to control confounding variables would further strengthen the evidence. Furthermore, exploring the neurobiological mechanisms linking dermatoglyphic patterns and pain perception, particularly through advances in neuroimaging and sensory analysis, may provide deeper insights into their clinical applicability.

## Conclusion:

Dermatoglyphic patterns demonstrated a significant association with pain perception in endodontic patients, with arch patterns indicating a higher pain response. Thus, dermatoglyphics as a non-invasive predictive marker for pain sensitivity, supporting its application in personalized endodontic care.

## Figures and Tables

**Table 1:** Association between dermatoglyphic pattern and pain score

**Pattern type**	**N**	**Mean**	**SD**	**95% Confidence Interval for Mean**		**F Value**	**P value**
				**Lower**	**Upper**		
Whorl	57	93.2941	33.0147	76.3195	110.2687	4.183	0.020*
Arch	63	118.8421	30.31	104.2332	133.4511		
Loop	80	93.1667	32.36566	79.4998	106.8335		
*Statistical significance
of p<0.05
